# A Study on the Preparation of Microbial and Nonstarch Polysaccharide Enzyme Synergistic Fermented Maize Cob Feed and Its Feeding Efficiency in Finishing Pigs

**DOI:** 10.1155/2020/8839148

**Published:** 2020-11-13

**Authors:** Biaosheng Lin, Jianbin Yan, Zhilong Zhong, Xintian Zheng

**Affiliations:** ^1^College of Life Science, Longyan University, Longyan 364012, China; ^2^Yi Zhitai Biotechnology (Longyan) Co., Ltd., Longyan 364012, China; ^3^Longyan Zhenggao Biotechnology Co., Ltd., Longyan 364012, China; ^4^Key Laboratory of Fujian Universities Preventive Veterinary Medicine and Biotechnology, Longyan University, Longyan 364012, China

## Abstract

1000 g maize cob mixed material was synergistically fermented by adding 2.5% composite probiotics and 0.06-0.08% NSP (nonstarch polysaccharide) enzyme to prepare fermented feed, and its effectiveness as feed for fattening pigs was investigated. The results showed that the appearance, texture, and nutrient quality of maize cobs significantly improved after fermentation, the total number of bacteria was 4.5 × 10^10^ CFU/g, and the protein content was 7.1%. Compared to the control group, the pigs in the 6% fermented maize cob feed experimental group showed significantly increased daily feed intake, daily weight gain, and nutrient digestion rate (*p* < 0.05) and reduced feed conversion ratio (*p* < 0.05). Most indicators including slaughter performance and meat quality significantly improved. In addition, beneficial bacteria including *Lactobacillus* in the intestines of the finishing pigs significantly increased, and pathogenic bacteria including *Escherichia coli* in the intestines and feces were found to be significantly reduced (*p* < 0.05). The intestinal crypt depth, VH/CD ratio, and ileal mucosal immunity of the finishing pigs also significantly improved (*p* < 0.05). The cytokine content and gene expression of sIgA, IL-8, and TNF-*α* were found to be significantly increased (*p* < 0.05). It could be concluded that the addition of 6% fermented maize cob feed to the diets of finishing pigs could promote their growth, improve their production performance and slaughter performance meat quality, and enhance their intestinal microecological balance and immunity.

## 1. Introduction

As a main feeding crop worldwide, maize has long been widely used in animal husbandry [1]. Maize cob is the central core after removing kernels from the maize ear. The annual output of corn in China exceeds 200 million tons, of which maize cob production accounts for approximately 10%, ultimately causing an extremely large output that exceeds 20 million tons [2]. With the development of science and technology, the field of maize cob deep processing has expanded continuously, and maize cobs have been processed into series of high value-added products, such as furfuryl alcohol, xylose, activated carbon, and glucose [3–5]. Maize cobs have also been widely used to produce ethanol [6–7], manufacture food packaging [8], extract oil [9], produce cultivation material for crops [10], and produce feedstuffs [11–13]; thus, maize cobs have high potential and value that should be fully accessed. Studies have shown that maize cobs primarily contain 32-36% cellulose, 35-40% hemicellulose, 17-20% lignin, and a small amount of ash and other components [14–15]. Their crude fiber content is high, and their palatability is poor. As the digestive utilization rate of direct feeding of animals is low, it is rarely used in animal production. Thus, preparing microbially fermented maize cob feeds is an economically feasible approach [16–17].

For many years, probiotics, such as *Lactobacillus*, yeasts, and *Bacillus subtilis*, have been widely used in feed fermentation [18–19]. In practice, however, simple microbial fermentation alone causes low protease content, which does not fulfill actual production needs. In addition, antagonism between microbial strains may also exist, which consequently affects the fermentation of products [20–21]. Synergistic microbial fermentation refers to fermentation that is subjected to enzymatic hydrolytic processing in conjunction with some amounts of probiotic bacteria. The addition of enzyme preparations overcomes the issue of insufficient enzyme production during fermentation by a single type of microorganisms and improves the utilization efficiency of feed macromolecules by the microorganisms [22–23]. In addition, a variety of organic acids and aroma substances that were produced by these probiotics during fermentation significantly improve the palatability of feed and regulate the intestinal health of animals [24–25].

Thus, this study combined the advantages of microbial probiotics in improving intestinal health and nonstarch polysaccharide (NSP) enzymes in degrading the principal nutritional components of maize cobs to develop a synergistic microbial fermented feed and investigate its feeding efficiency in finishing pigs. High-quality mixed feed suitable for finishing pigs was developed, thereby transforming waste into a valuable resource, extending the industrial chain of agricultural byproducts and waste materials, such as maize cobs, and providing a basis for its application in animal husbandry.

## 2. Materials and Methods

### 2.1. Materials

Maize cobs (Denghai 605) were purchased from market (Jinxiu Agricultural and Sideline Products Processing Co., Ltd., Qihe, China), and its moldy and rotten parts were removed, dried at 60~70°C for 4 h, and then crushed by 50~100-mesh sieve. Freeze-dried bacterium blended powder containing 6.2 ~ 7.7 × 10^9^ CFU/g of *Lactobacillus fermentum*, 1.3 × 4.6 × 10^8^ CFU/g of *Saccharomyces cerevisiae*, and 2.5 ~ 4.2 × 10^8^ CFU/g of *Bacillus subtilis* and NSP enzyme containing 10000~20000 U/g activity of xylanase, 1000~1500 U/g activity of *β*-glucanase, 200~300 U/g activity of mannanase, 2000~4000 U/g activity of cellulase, and 100~300 U/g of pectinase were all provided by Fujian Longyan Jinhe Animal Feed Co., Ltd., China. Fermenter and select PE (polyethylene) membrane with unidirectional permeability holes (purchased from the market) were 5~7-layer thick and have permeability hole diameter 9-12 mm, oxygen permeation 2.25~4.42 cm^3^/m^2^.d.bar, and exhaust pressure 678~750 mmH_2_O. Duroc-Landrace-Yorkshire fattening pigs were purchased and housed in Yi Zhitai Biotechnology (Longyan) Co., Ltd. (Longyan, China).

### 2.2. Medium

Maize cob fermentation medium (1000 g) consisted of 600 g maize cob, 30 g corn flour, 15 g brown sugar, 15 g compound multivitamins, 340 mL water, and pH 6.0~6.5. Among them, compound multivitamin composition per kilogram is as follows: vitamin A 4000000 IU, vitamin D3 1200000 IU, vitamin E 30000 IU, vitamin K_3_ 800 mg, vitamin B_1_ 1000 mg, vitamin B_2_ 3200 mg, vitamin B_6_ 800 mg, vitamin B_12_ 24 mg, D-pantothenic acid 14400 mg, folic acid 2800 mg, and niacin 24000 mg. In addition, NSP enzyme was added for another 0.6-0.8 g during fermentation.

### 2.3. Preparation of Fermented Maize Cob Feed

25 g of bacterial powder was weighed for every 1000 g of material to be fermented. The bacterial powder was then activated by adding warm water at a temperature of 25-30°C and stirred thoroughly. The amount of water used was according to the manufacturer's formulation, and the warm water contained the amount of brown sugar specified in the formulation. The brown sugar was thoroughly mixed with the warm water described above. Raw materials were set up as specified by the formulation, mixed thoroughly, loaded into the fermenter, connected with the activated bacterial mixture, and mixed once again for closed fermentation. Fermentation conditions were as follows: fermentation temperature of 25-30°C and resting time of 5-7 d. The fermented material was suitable for feeding when its color became deeper and darker and developed a clear scent.

### 2.4. Determination of Fermented Feed Product Performance

The appearance of the product before and after fermentation, microbial strain content, and nutrient composition were analyzed. The microbial strain contents were measured by live bacteria plating. Crude protein, dry matter, crude ash, neutral detergent fiber, acid detergent fiber, crude fat, reducing sugar, calcium, phosphorus, and other nutrient components were analyzed by referring to conventional feed analysis methods [26].

### 2.5. Measurement Indices and Methods for Fermented Maize Cob Feed for Finishing Pigs

For the growth performance and nutrient consumption, a total of 200 healthy Duroc × (Landrace × Yorkshire) three-way crossbred finishing pigs weighing 60.12 ± 0.75 kg were selected and randomly divided into 4 groups. Each group included 5 repetitions, and each repetition included 10 pigs (of similar weight) that were divided evenly between males and females for 60-120 kg feeding tests. The 4 groups included the control group and 3 experimental groups. The control group was fed a basic diet (Table 1, according to the NRC (2012) nutrient requirements for finishing pig), and the experimental groups were fed the basic diet supplemented with 4, 6, and 8% fermented maize cob feed—replacing the energy components, such as corn and soybean meal, of the basic diet. The addition of concentration ratios (4%, 6%, and 8%) was based on the addition ratio of conventional fermented feed in livestock and poultry animals and the results of prefeeding in the early stage of this experiment [27–28]. The growth performance and nutrient digestibility of each group of finishing pigs were analyzed in a finishing pig house with relatively stable and controlled conditions. Further, the growth performance indices of the average daily weight gain, feed intake, and feed conversion ratio in finishing pigs that were fed fermented maize cob, rather than conventional feed, were investigated.

For the evaluation of the nutrient digestibility effects, titanium dioxide (TiO_2_) was used as an external index for digestion tests. 0.1% TiO_2_ was added to the experimental groups that were fed fermented feed, and both feed and fecal samples were collected from each group after prefeeding for 5 days. Gross energy (GE), dry matter (DM), crude protein (CP), ether extract (EE), crude ash (Ash), calcium levels (Ca), and phosphorus levels (P) in the samples were analyzed and determined to evaluate the effects of fermented maize cob feed on nutrient digestibility in finishing pigs. Ti contents were determined as described by Morgan et al. [29]. Lastly, the nutrient consumption rate (%) was equal to the following: [1 − (Ticontentsinfeedsamples/Ticontentsinfecalsamples) × (nutrientcontentsinfecalsamples/nutrientcontentsinfeedsamples)] × 100.

For the slaughter performance and meat quality, after testing, five finishing pigs from each experimental group were randomly selected for slaughter, and the slaughter performance, meat quality, muscle fat levels, and fatty acid levels of the pork were evaluated. Each index was measured as described by Panella-Riera et al. [30]. All pigs were slaughtered with a normal humane procedure, and all efforts were made to minimize suffering. The pigs were euthanized by electric shock and then dehaired, and the carcasses were dissected.

For intestinal performance and ileal mucosal immunity, after testing, fecal samples were aseptically collected from the rectum of the finishing pigs before slaughter, and the total bacteria and *E. coli* counts of the collected samples were measured (plate colony counting method, the same as below). The morphology of the intestinal tissue was examined at the time of slaughter, and the duodenum, jejunum, ileum, and cecum were isolated. Of these, the duodenum, jejunum, and ileum were stored in 10% neutral formalin buffer solution and frozen sections were prepared as described by Hu et al. [31]. Hematoxylin-eosin (HE) staining was then performed. The villus height (VH), crypt depth (CD), and VH/CD ratio were calculated. Chyme from the ileum and cecum was collected for microbial flora determination. Approximately 1.5 cm of the proximal distal ileum was treated with normal saline, frozen in liquid nitrogen, and stored in a -80°C freezer. The double-antibody sandwich enzyme-linked immunosorbent assay (ELISA) method was used to measure the contents of secreted immunoglobulin A (sIgA), interleukin-8 (IL-8), and tumor necrosis factor-*α* (TNF-*α*) in the intestinal tissue supernatant. All kits were purchased from Nanjing Jiancheng Bioengineering Institute, and a real-time PCR assay was used to measure the mRNA expression of IL-8 and TNF-*α*. The real-time PCR reaction composition was as follows: 10.0 *μ*L of 2× Master Mix (Beijing Tiangen Biochemical Technology Co., Ltd.), 0.5 *μ*L of primer F (10 *μ*M), 0.5 *μ*L of primer R (10 *μ*M), q.s. to a total volume of 20 *μ*L with diethyl pyrocarbonate- (DEPC-) treated water, and 1.2 *μ*L of cDNA (30 ng/*μ*L). Primer pairs for each factor are shown in Table 2 [32]. The reaction procedure was as follows: 95°C for 30 s, 95°C for 5 s, and 60°C for 35 s; 40 cycles. Further, the melting curve analysis was based on automated fluorescence measurements as follows: 60°C for 60 s and 95°C for 15 s (60°C-95°C). The 2^-*ΔΔ*CT^ method was used to calculate the expression of each factor in the experimental groups with the addition of different amounts of fermented maize cob feed relative to the control group. 18S RNA was used as the internal reference gene [33].

### 2.6. Statistical Analysis

All the data were sorted by Excel software, and then, one-way ANOVA program in SPSS software was used for single-factor ANOVA analysis, Waller-Duncan program for multiple comparison between groups. All data in test results were represented by the mean ± SD, and means were considered different when *p* < 0.05.

## 3. Results

### 3.1. Measurement Results of Fermented Maize Cob Feed Product Performance

Results of the performance measurements of maize cob before and following fermentation are shown in Table 3. Maize cobs exhibited a deeper color following fermentation, which produces a transparent wine-like and lactic acid scent with a clear color and texture change. Compared to the levels before fermentation, the contents of crude protein, calcium, and phosphorus in maize cobs increased after fermentation, whereas dry matter, crude fat, neutral detergent fiber, acid detergent fiber, crude fat, and reducing sugar contents decreased. Compared to single bacterial fermentation, synergistic microbial fermentation of maize cob with the addition of NSP enzymes significantly increased each microbial strain population, fiber degradation, and protein contents (*p* < 0.05); the residual contents of dry matter, crude ash, and reducing sugar decreased. The results showed that the addition of NSP enzymes could increase the utilization efficiency of maize cob macromolecules and significantly improved the protein conversion efficiency. Furthermore, this addition also provided more energy for microbial strain growth and significantly increased the number of each microbial strain.

### 3.2. Effects of Fermented Maize Cob Feed on Growth Performance and Nutrient Consumption of Finishing Pigs

As shown in Table 4, the daily feed intake significantly increased in every experimental group as the amount of fermented feed increased (*p* < 0.05), compared to the control group. Daily weight gain significantly increased (*p* < 0.05), whereas the feed conversion ratio was found to be reduced. When the amount of additive was 6%, the daily weight gain and feed conversion ratio was significantly improved compared to that of the control and 4% additive groups (*p* < 0.05), with no significant differences compared to the 8% additive group.

Table 5 shows that gross energy, dry matter, organic matter, crude protein, crude fat, calcium levels, phosphorus levels, and other nutrient digestibility indices of fermented maize cob feed significantly increased compared to the control group (*p* < 0.05). Among these indices, when the amount of additive was 6%, many nutrient digestibility indices were significantly higher than in the 4% additive group (*p* < 0.05), with no significant differences compared to the 8% additive group.

### 3.3. Effects of Fermented Maize Cob Feed on Slaughter Performance and Meat Quality of Finishing Pigs

Table 6 shows that the addition of different proportions of fermented maize cob feed exhibited positive effects on most indices, such as slaughter performance, meat quality, muscle fat levels, and fatty acid levels, compared to those of the control group, but some indices (dressed weight, dressing percentage, tenth rib fat thickness, and percent lean) were not significantly improved with increased additive. Overall, the 6% additive condition significantly improved the slaughter performance and meat quality of finishing pigs (*p* < 0.05).

### 3.4. Effects of Maize Cob Fermented Feed on Intestinal Performance and Ileal Mucosal Immunity in Finishing Pigs

**Figure 1:**
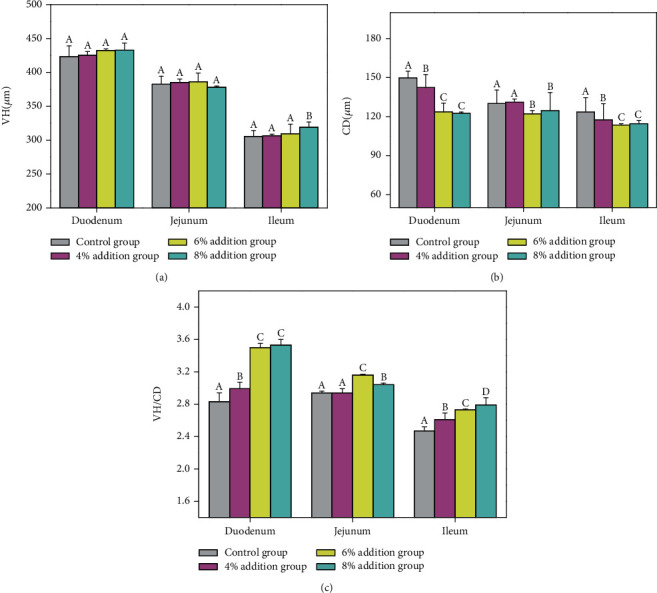
Effects of fermented maize cob feed on intestinal morphological structure of finishing pigs: (a) villus height; (b) crypt depth; (c) villus height/crypt depth. Value columns with different letters mean significant difference (*p* < 0.05), the same as below.

**Figure 2:**
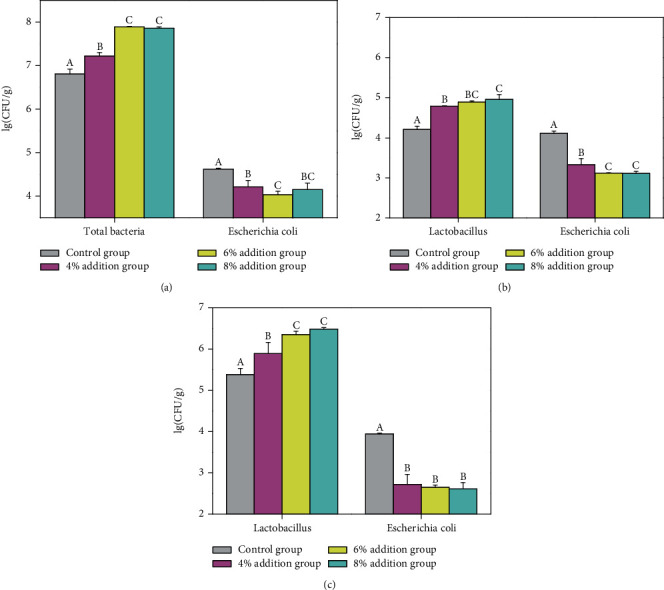
Effect of fermented maize cob feed on feces and intestinal microflora of finishing pigs: (a) changes in the microbial flora of feces in different test groups; (b) changes in the microbial flora of the ileum in different test groups; (c) changes in the microbial flora of the cecum in different test groups.

**Figure 3:**
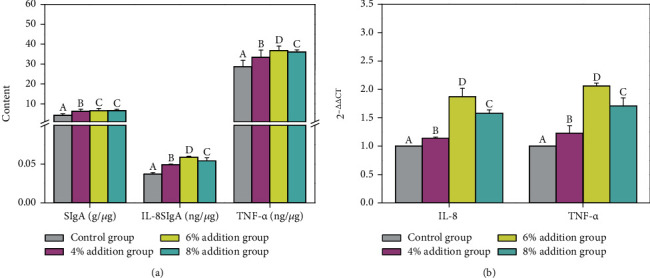
Effect of fermented maize cob feed on ileal mucosal immunity of finishing pigs: (a) changes in cytokine content in different test groups; (b) changes in gene expression of immune factors in different test groups.

Figure 1(a) shows that different proportions of fermented maize cob feed did not significantly increase the villus height of finishing pigs compared to the control group, although crypt depth (Figure 1(b)) and VH/CD ratio (Figure 1(c)) were found to be significantly decreased and increased, respectively, as the additive amount increased (*p* < 0.05). Further, intestinal morphology and structure were improved. The addition of fermented maize cob feed exhibited significant effects on the feces and intestinal microorganisms of the finishing pigs (*p* < 0.05). Compared to the control group, the microbial florae in the feces of finishing pigs increased and the number of *E. coli* significantly decreased (*p* < 0.05) as the amount of additive increased (Figure 2(a)), while *Lactobacillus* content in the ileum and cecum significantly increased and the number of *E. coli* decreased (*p* < 0.05) (Figures 2(b) and 2(c)). Fermented maize cob feed also significantly improved the ileum mucosal immunity of the finishing pigs (*p* < 0.05) (Figure 3). Compared to the control group, the cytokine contents and expression of the corresponding immune factor genes in each experimental group increased as the amount of additive increased—all of which reached significance (*p* < 0.05).

## 4. Discussion

Synergistic microbial fermentation techniques connect the entire process of feed fermentation, processing, and production. The combined action of microbial probiotics and enzymatic hydrolysis technology was the biggest technological breakthrough in feed fermentation and was important for the future development of biological feed [34]. The present study demonstrated that the addition of different proportions of synergistic microbial fermented maize cob feed promoted the growth, nutrient consumption, slaughter performance, and the overall intestinal health of finishing pigs. It could also replace the energy component of the basic diet part in “NRC (2012) nutrient requirements for finishing pig,” and these effects were significantly enhanced as the proportion of additive increased until an equilibrium was maintained at a peak value.

The nutrient consumption rate of livestock feed was an important index to measure the digestive utilization of livestock animals and to evaluate the nutritional value of feed [35]. NSP, the principal component of plant-derived cell walls, was not easily digested or utilized by monogastric animals. In addition, water-soluble nonstarch polysaccharides (e.g., arabinoxylan and *β*-glucan) are highly viscous and can increase the chyme viscosity in the intestines of animals, which blocks interactions between nutrients in feed and digestive juices and affects the digestion of nutrients. The addition of NSP enzymes can eliminate or reduce the adverse effects of NSP [36–37]. NSP enzymes can degrade plant cell walls, cleave internal soluble nonstarch polysaccharides, and promote the release of nutrients bound in cell walls. Reducing the viscosity of the contents of the intestinal tract was beneficial for interactions between nutrients and enzymes and improved digestion rates [38]. On the other hand, composite probiotics in fermented feed can decompose macromolecular substances that were difficult for livestock and poultry to digest into small molecule nutrients, such as small peptides, glucose, amino acids, and vitamins, which were easily digested and absorbed by the body of the animal [39]. Additionally, lactic acid and ethanol that were secreted by probiotics during their growth also improve the palatability of feed and stimulate increased feed intake by pigs [40]. In the present study, probiotics, such as lactic acid bacteria and yeasts contained in the fermented maize cob feed, underwent synergistic fermentation with NSP enzymes. Finally, the nutrient consumption rate and nutrient digestion and absorption in the fermented maize cob feed experimental group of finishing pigs significantly increased, and the production performance of the finishing pigs (feed conversion ratio, daily weight gain) also significantly increased.

Slaughter performance and meat quality were the main indices that evaluate the performance of livestock products [41]. Adding fermented maize cob feed promotes absorption of dietary nutrients, accelerated growth rate, and increased the dressed weight and dressing percentage of finishing pigs. However, when a high proportion (8%) of fermented maize cob feed was added, the energy intake became too high, and rapid back fat accumulation occurred, thus reducing percent lean and slaughter quality of the finishing pigs. Studies had shown that the metabolites of microorganisms can increase the cytoplasmic concentration in pork cells, increase the ability of the pork to absorb water, and reduce the drip loss of pork [42]. The present study also found that fermented maize cob feed can reduce the drip loss of pork, which may be due to enhanced enzymatic hydrolysis of nutrients in the fermented feed and increased production of metabolites from microbial growth. Moreover, the probiotics in fermented maize cob feed can promote the conversion of fat into tissue by the liver and oxidative degradation, reduce fatty acid content in the liver, and simultaneously provide energy for protein synthesis, thus improving meat quality. In addition, the scent of the pork was affected by intermuscular fat and fatty acid composition. Cameron et al. and Makoto *et al.* found relationships between pork eating quality (tenderness, juiciness, and flavor) and muscle fatty acid composition [43–44]. In the present study, as the content of saturated fatty acids and monounsaturated fatty acids in each fermented maize cob feed experimental group increased, pork eating quality increased to some degree. Conversely, increased polyunsaturated fatty acid content led to decreased eating quality.

The balance of the microecological system in animal intestines plays an important role in improving growth rate, promoting immune system development, maintaining normal immune function, defending against pathogen invasion, and reducing disease occurrence in animals [45]. The present study found that the addition of fermented maize cob feed to the diet of finishing pigs resulted in abundant probiotics that rapidly occupied the ecological niches in the intestines of the pigs, thereby establishing growth dominance, significantly increasing the number of lactic acid bacteria in the intestines, and reducing the amount of *E. coli* in the intestines and feces. This overall improved the microecological balance in the intestines of the pig and improved physical immunity.

Morphological structural integrity, villus height, and crypt depth of the small intestines were important criteria to measure the health of the animal and its ability to digest and absorb nutrients [46]. Studies of the intestinal surface suggested that longer villi were related to the improved ability of the small intestines to absorb nutrients, shallower crypt depth related to the improved small intestinal secretion activity, and greater villus height/crypt depth ratio related to the larger intestinal lining area and higher digestive capacity [47]. In the present study, fermented maize cob feed contained yeast, of which its cell wall contains *β*-glucan and mannan, which reduce the binding of the gastrointestinal tract mucosa of pigs to antigens via the adsorption, phagocytosis, destruction, and absorption of invading bacteria. This consequently protects the gastrointestinal tract mucosa from damage, protects the morphological structural integrity of the small intestines, and promotes small intestine development and significant improvement in crypt depth and the VH/CD ratio.

sIgA was an important effector molecule in the intestinal mucosa that can regulate intestinal microorganisms and neutralize toxins [48]. Cytokines—such as IL-8 and TNF-*α*—were important signaling molecules in the immune system [49]. Probiotics stimulate the expression and secretion of proinflammatory factors in the intestinal immune cells of pigs and regulate the host immune function towards a more stable state [50–51]. In the present study, fermented maize cob feed significantly increased the content of sIgA cytokines and IL-8 and TNF-*α* immune factors in the ileum of finishing pigs, which may be due to the entry of probiotics in maize cobs into the intestines of piglets as antigens to stimulate mucosa and promote B cell proliferation and differentiation in plasma cells, which thus secretes a large amount of sIgA to improve mucosal immune function and improve disease resistance. On the other hand, the added exogenous microorganisms were recognized by the body of the animal, which stimulates the mucosa to produce a mild inflammatory response. This increased the expression of proinflammatory factors in small intestinal mucosae, which thus increased anti-inflammatory factors in the intestines, enhanced the immune function of the ileal mucosae, and improved the anti-infective capacity of the body.

However, the addition of more probiotic-fermented feed did not always lead to improvements, since an optimal dose exists [52, 53]. The present study found that the addition of beyond 6% fermented maize cob feed slowed increases in growth performance and other growth- and production-related indices in finishing pigs. The basic reason behind that is that as the amount of probiotic-fermented feed increased, the stress on the intestines of the pig increased and immune factor content and expression reduced, which may eventually lead to faster fat deposition rate and lower meat quality. Given the economic benefits of overall feeding costs, the addition of 6% fermented maize cob feed was selected as an optimal dosage to feed finishing pigs.

In the present study, a combined probiotic and NSP enzyme fermentation technique was employed to prepare a fermented maize cob feed, which enhanced the degradation of maize cob composition and improved its nutritional value. The addition of 6% fermented maize cob feed to the diets of finishing pigs promoted their growth and improved their production performance, slaughter performance, and meat quality. In addition, their intestinal microecological balance was improved and their immunity was enhanced, which provides a theoretical basis and practical examples for comprehensive utilization of maize cobs and the development of microbe-fermented feed preparation techniques.

## Figures and Tables

**Table 1:** Components and nutritional level of forages for tested pigs.

Components of basic diet (%)		Nutritional level	
Corn	53~68	Energy (MJ·kg^−1^)	15.68~15.97
Bean cake CP (44%)	22~36	Crude protein (%)	12.65~19.38
Rice bran	2~6	Dry matter (%)	86.25~88.97
Complex premix compound	2~4	Crude fiber (%)	2.45~2.86
Total	100	Ca (%)	0.56~0.72
		Available phosphorus (%)	0.24~0.31
		Lysine (%)	0.85~1.00

**Table 2:** Primer sequences for real-time PCR.

Gene	Primer sequence (5′- -3′)	Fragment size (bp)	Tm (°C)
IL-8	F: TTCGATGCCAGTGCATAAATA	176	60
	R: CTGTACAACCTTCTGCACCCA		
TNF-*α*	F: CCAATGGCAGAGTGGGTATG	116	60
	R: TGAAGAGGACCTGGGAGTAG		

**Table 3:** Determination of maize cob composition before and after fermentation.

Items		Before processing	After processing (added bacteria but no NSP enzyme)	After processing (added bacteria and NSP enzyme)
Visual evaluation		Yellowish brown, powdery, tough, uniform tissue, no obvious fragrance, slightly dry	Brown, with a certain lactic acid and wine flavor, soft texture, loose, slightly moist	Dark brown, more obvious lactic acid and wine flavor, soft smell, not pungent, loose, soft, and moist
Bacteria content		—	Total bacteria count: 3.6 × 10^10^ CFU/g, containing 6.8 × 10^6^ CFU/g *of Lactobacillus fermentum*, 5.1 × 10^6^ CFU/g of *Saccharomyces cerevisiae*, 3.5 × 10^10^ CFU/g of *Bacillus subtilis*	Total bacteria count: 4.5 × 10^10^ CFU/g, containing 7.2 × 10^6^ CFU/g *of Lactobacillus fermentum*, 7.4 × 10^6^ CFU/g of *Saccharomyces cerevisiae*, 4.4 × 10^10^ CFU/g of *Bacillus subtilis*
Nutrient composition (%)	Crude protein	3.4 ± 0.09^a^	5.9 ± 0.13^b^	7.1 ± 0.15^c^
	Dry matter	97.1 ± 1.89^a^	96.5 ± 2.21^a^	92.9 ± 2.16^b^
	Crude ash	2.9 ± 0.01^a^	2.1 ± 0.12^b^	1.8 ± 0.08^b^
	Neutral washing fiber	88.5 ± 0.87^a^	70.3 ± 1.78^b^	61.5 ± 2.21^c^
	Acid detergent fiber	42.6 ± 2.52^a^	42.2 ± 2.03^a^	39.8 ± 1.12^b^
	Crude fat	0.52 ± 0.12^a^	0.34 ± 0.11^b^	0.29 ± 0.09^b^
	Reducing sugar	2.15 ± 0.11^a^	1.42 ± 0.12^b^	1.18 ± 0.13^c^
	Ca	0.12 ± 0.02^a^	0.28 ± 0.05^b^	0.33 ± 0.03*b*
	P	0.04 ± 0.001^a^	0.05 ± 0.002^b^	0.06 ± 0.001^b^

Note:“-” means no value, all the data in the table were the determination results of corncob before and after fermentation at different batches, and five batches were determined. In the shoulder markers of peer data, The same letters indicate no significant difference (*p* > 0.05), and different letters indicate significant difference (*p* < 0.05), the same as below.

**Table 4:** Effect of fermented maize cob feed on growth performance of finishing pigs.

Group	Control group	Test group		
		4% additive group	6% additive group	8% additive group
Initial weight (kg)	59.93 ± 3.41^a^	60.21 ± 4.21^a^	60.13 ± 1.54^a^	60.25 ± 2.24^a^
Final weight (kg)	118.69 ± 2.65^a^	120.32 ± 3.21^a^	121.36 ± 2.65^a^	121.63 ± 1.65^a^
Average daily gain (g/d)	691.29 ± 20.12^a^	707.18 ± 25.36^a^	720.35 ± 22.12^b^	722.12 ± 18.69^b^
Average daily intake (g/d)	2135.36 ± 56.32^a^	2145.98 ± 74.25^c^	2140.36 ± 36.68^b^	2141.32 ± 56.32^b^
Feed conversion ratio	3.09 ± 0.07^a^	3.03 ± 0.05^b^	2.97 ± 0.06^c^	2.97 ± 0.05^c^

Note: the feeding time was July~October 2019, the pretest period of feeding the basic diet before the formal test was 3 d, and then, the formal feeding test was carried out for 85 d.

**Table 5:** Effect of fermented maize cob feed on nutrient digestibility of finishing pigs.

Group	Control group	Test group		
		4% additive group	6% additive group	8% additive group
Total energy (%)	81.92 ± 5.21^a^	83.31 ± 3.12^b^	85.72 ± 3.42^c^	86.04 ± 6.41^c^
Dry matter (%)	82.60 ± 2.36^a^	84.73 ± 5.62^b^	86.71 ± 2.45^c^	84.68 ± 4.65^b^
Organic matter (%)	85.85 ± 5.65^a^	87.82 ± 3.25^b^	87.80 ± 4.52^b^	89.32 ± 2.98^c^
Crude protein (%)	77.11 ± 4.56^a^	80.44 ± 2.32^b^	82.33 ± 4.56^c^	82.34 ± 4.68^c^
Crude fat (%)	33.62 ± 1.23^a^	61.79 ± 2.21^b^	63.15 ± 1.87^c^	62.99 ± 2.12^c^
Ca (%)	35.94 ± 1.12^a^	43.35 ± 1.25^b^	44.02 ± 1.68^bc^	44.93 ± 1.59^c^
P (%)	51.01 ± 1.67^a^	54.20 ± 2.25^b^	56.13 ± 2.20^c^	55.99 ± 4.36^bc^

**Table 6:** Effect of fermented maize cob feed on slaughter performance and meat quality of finishing pigs.

Group		Control group	Test group		
			4% additive group	6% additive group	8% additive group
Slaughter performance	Weight before slaughter (kg)	118.69 ± 2.65^a^	120.32 ± 3.21^a^	121.36 ± 2.65^a^	121.63 ± 1.65^a^
	Carcass weight (kg)	84.41 ± 2.21^a^	86.30 ± 2.35^ab^	88.21 ± 1.65^b^	87.01 ± 2.03^ab^
	Dressing percentage (%)	71.12 ± 1.56^a^	72.56 ± 2.31^b^	72.68 ± 2.14^b^	71.54 ± 1.89^a^
	10th ribbed back fat thick (cm)	2.80 ± 0.32^a^	2.78 ± 0.24^ab^	2.76 ± 0.16^b^	2.79 ± 0.30^ab^
	10th costal muscle area (cm^2^)	37.45 ± 1.13^a^	37.94 ± 1.36^b^	38.14 ± 1.34^c^	38.10 ± 1.42^c^
	Thin meat rate (%)	55.42 ± 1.45^a^	56.01 ± 2.01^ab^	56.32 ± 1.36^b^	55.69 ± 1.37^a^
Meat quality	Meat color score	2.12 ± 0.13^a^	3.11 ± 0.16^b^	3.20 ± 0.35^c^	3.22 ± 0.21^c^
	Marbling score	2.32 ± 0.21^a^	3.16 ± 0.21^b^	3.26 ± 0.26^c^	3.24 ± 0.16^bc^
	pH_45min_	6.12 ± 0.36^a^	6.15 ± 0.31^a^	6.25 ± 0.29^b^	6.25 ± 0.38^b^
	pH_24h_	5.51 ± 0.29^a^	5.52 ± 0.27^a^	5.57 ± 0.31^b^	5.57 ± 0.39^b^
	Tenderness (N)	30.52 ± 1.03^a^	23.44 ± 0.98^b^	23.15 ± 1.12^c^	23.21 ± 1.03^c^
	Water loss rate (%)	42.36 ± 1.13^a^	42.22 ± 1.12^b^	42.12 ± 1.25^c^	42.20 ± 1.20^bc^
	Drip loss (%)	2.71 ± 0.16^a^	2.68 ± 0.21^a^	2.63 ± 0.18^b^	2.65 ± 0.22^b^
Muscle fat levels and fatty acid levels	Meat fat (%)	2.31 ± 0.26^a^	2.35 ± 0.21^ab^	2.38 ± 0.16^b^	2.44 ± 0.19^c^
	Monounsaturated fatty acid (%)	43.36 ± 1.17^a^	45.21 ± 1.16^b^	45.32 ± 1.05^bc^	45.38 ± 1.21^c^
	Polyunsaturated fatty acid (%)	11.15 ± 0.21^a^	10.65 ± 0.32^ab^	10.23 ± 0.25^bc^	10.02 ± 0.29^c^
	Unsaturated fatty acid (%)	54.51 ± 1.36^a^	55.86 ± 1.38^b^	55.55 ± 1.25^d^	55.40 ± 1.28^c^
	Saturated fatty acid (%)	40.12 ± 1.14^a^	40.20 ± 1.03^ab^	40.22 ± 0.89^b^	40.23 ± 0.97^b^

## Data Availability

All data are fully available without restriction, and all relevant data are within the paper.
